# Effects of modern dance programs on improving health-related physical fitness in girls

**DOI:** 10.3389/fpubh.2024.1425974

**Published:** 2024-11-21

**Authors:** Aleksandra Ilić, Dragan Marinkovic, Romina Herodek, Jadranka Vlašić, Saša Jovanović

**Affiliations:** ^1^Faculty of Sport and Physical Education, University of Novi Sad, Novi Sad, Serbia; ^2^Faculty of Sport and Physical Education, University of Nis, Nis, Serbia; ^3^Faculty of Kinesiology, University of Zagreb, Zagreb, Croatia; ^4^Faculty of Physical Education and Sport, University of Banja Luka, Banja Luka, Bosnia and Herzegovina

**Keywords:** modern dance, physical activity, physical fitness, ALPHA battery of tests, younger school age

## Abstract

**Introduction:**

As creative physical activity influences many aspects of child development—including physical, social, and emotional wellbeing—the effects of two experimental modern dance programs on the development of health-related physical fitness in young school-aged girls were assessed in this longitudinal study.

**Materials and methods:**

The study sample comprised 203 girls aged 7–11 years, 102 of whom (the control group) were recruited from the elementary school “Vuk Karadžić” from Bačka Palanka, while Experimental Group 1 (*n* = 51) and Experimental Group 2 (*n* = 50) respectively consisted of girls who have been involved in a modern dance program for no longer than 1 year and at least 2 years. The participants were also divided into AGE 1 (7–9 years, *n* = 103) and AGE 2 (9–11 years, *n* = 100) groups to assess the influence of age on the treatment effect. During the six-month experimental period, Experimental Group 1 met three times a week while Experimental Group 2 had five weekly training sessions. All participants were subjected to the extended ALPHA battery of tests both at baseline and at the end of the study.

**Results:**

The Experimental Group 1 and Experimental Group 2 results showed a statistically significant difference (*p* = 0.005) between the initial and final measurements, suggesting that participation in a modern dance program had a positive impact on the transformation of morphological characteristics by increasing muscle mass and reducing subcutaneous fat. In addition, both experimental groups had a greater muscle strength, muscle endurance, and aerobic fitness at the end of the treatment.

**Conclusion:**

The study findings support the view that health-related physical fitness in young school-aged children can be improved through modern dance.

## 1 Introduction

Changes in lifestyle and occupational habits that have taken place in recent decades have resulted in declining physical activity levels (1–3). This is particularly problematic for children and adolescents, as sedentary lifestyle during this crucial developmental stage can have important ramifications for physical and mental health. According to the World Health Organization (4) and other renowned institutions (5, 6), youth should engage in moderate-to-vigorous physical activity for at least 60 min each day to maintain optimal physical fitness (7).

Physical fitness is defined as the physiological state of health and functional capacity that facilitates satisfaction of all daily living requirements (8). It is reflected in the state of all body functions—musculoskeletal, cardiorespiratory, hemato-circulatory, psycho-neurological, metabolic, and endocrine—and can be affected by the degree and scope of daily physical activities (9).

While the physical fitness level depends on many factors, including genetic characteristics (10), it also plays a role in numerous psychological indicators (11–13). According to the extant research, fitter children tend to feel less alone and are thus less prone to depression, while exhibiting stronger social, cognitive, and athletic competencies as well as self-esteem (14).

In this context, it is important to differentiate between performance-and health-related physical fitness, as the former refers to the preparedness for sports competitions and professional work, while the latter pertains to general health and the risk of developing chronic diseases (15). Ample body of evidence shows that individuals that regularly participate in sports, recreation, and other physical activity modes are more likely to maintain an optimal level of health-related fitness (16), while lowering their risk of illness and injury and increasing their resilience to fatigue (17). Increasingly, explosive strength is being recognized as a part of health-related physical fitness (18).

As explosive strength is important for dynamic dance movements, including jumps and turns, regular participation in modern dance classes is posited to contribute to young children’s health. This type of strength improves flexibility and coordination, as well as general physical fitness. Additionally, combining dance with explosive strength training can increase cardiovascular endurance, improve body composition, and enhance athletic performance. These elements are especially crucial for young dancers because they can encourage a lifetime commitment to fitness. Compared to ballet dancers, both males and females that regularly partake in modern dance are typically stronger and can in many cases easily compare in strength with other athletes (19).

Determining the difference between activities and exercises designed to build muscle strength and those focusing on muscle endurance or explosive strength in real life can be challenging, especially for younger school-aged children. Thus, when working with this age group, focus tends to be on “muscular fitness,” including all three muscle components. As aerobic fitness directly indicates a person’s physiological state, it is a particularly important aspect of health-related fitness, which also indicates the cardiovascular and respiratory systems’ combined capacity to provide oxygen to the body during extended, intense activity (20). In addition to improving muscle strength and explosiveness, well-planned and supervised resistance exercises aimed at the development of muscular fitness in children and adolescents can lower the risk of cardiovascular diseases, improve motor skills and sports performance, elevate resistance to sports injuries, enhance psychosocial wellbeing, and encourage the development of positive lifestyle habits (21).

Physical fitness has long been a reliable indicator of long-term health in this age group (9, 22), and is regarded as an essential health measure (23, 24). Accordingly, it is disconcerting to note that children’s reported levels of physical activity and physical fitness have declined over the past few decades (25), while prevalence of obesity has increased. This significant global health problem is associated with modifications to somatotype and body composition (26), which may be detrimental to physical fitness (27).

While the causes and determinants of changes in physical fitness are still not fully understood, weight and body mass index (BMI) are typically evaluated, among other health-related parameters (28). Additionally, extant research has demonstrated a negative correlation between aerobic fitness and overweight status as well as poor health in adolescents (29).

As cardiorespiratory fitness in the general population has been on a steady decline (30), modern dance is seen as a viable means of arresting or even reversing this negative trend. As one of the most attractive forms of physical activity, it can be performed by individuals of all ages (31). Dance improves muscle strength, cardiovascular endurance, and joint flexibility, while having beneficial effects on physical condition by increasing muscle mass, reducing subcutaneous fat tissue, and improving the functioning of cardiovascular, respiratory, and metabolic systems (32). As shown by Duberg et al. (33), dance also offers numerous mental health benefits by reducing stress and boosting self-confidence, making it a great way to improve overall wellbeing.

While research on the impact of dance on various anthropological characteristics remains limited, previous studies have primarily focused on modern dance and health-related physical fitness on professional levels and young adults. Additionally, this study examines the effects of different modern dance programs specifically designed for girls in middle childhood. This gap in the literature has inspired the current research. The work presented here is important because it broadens our knowledge of the connection between physical activity, health, and child development and because it may inspire further research in this field. Enhancing children’s health and wellbeing through the development of fundamental motor skills and physical activity promotion are its two potential contributions. Theoretically, it advances the current understanding of the role of modern dance in girls’ physical fitness by identifying the essential aspects of health and physical form that can be enhanced through regular training. Its practical value stems from the beneficial suggestions for the design of physical education programs in schools with the aim of increasing students’ motivation and involvement levels. The obtained findings can also be implemented in dance schools to improve the class content effectiveness. Therefore, the main study’s purpose was to determine the effects of two experimental modern dance programs on the development and improvement of health-related physical fitness in younger school-aged girls. It was guided by the hypothesis that, in comparison to girls who did not engage in this kind of physical activity, girls who took part in an experimental modern dance program would show significantly greater progress in the development of morphological characteristics, motor abilities, and every aspect of physical fitness related to health.

## 2 Materials and methods

### 2.1 Participants

The study sample consisted of female pupils from “Vuk Karadžić” elementary school in Bačka Palanka (the control group) and girls involved in various modern dance programs at the “PLAY DANCE STUDIO” dance club in Novi Sad who were segregated into two experimental groups based on their experience/skill level. The study eligibility criteria were as follows:

- No injuries in the past 6 months;
- Absence of any medical conditions, including COVID-19;
- No participation in a scheduled physical activity in the previous 3 months;
- Completion of at least 80% of all training sessions for the experimental groups;
- Signed consent forms from parents/legal guardians. Participants were ineligible for the study if they met any of the following criteria:
- A history of neurological or musculoskeletal disorders;
- Clinical conditions that could impact balance, such as motor disorders, diabetes, heart disease, stroke, vision problems, thyroid issues, or issues with nerves or blood vessels.

The final sample for this research included 203 female participants (age: 8.63 ± 1.13 years; height: 132.56 ± 0.09 cm; weight: 30.24 ± 7.85 kg), who were assigned to one of the following three groups: (1) the control group (*n* = 102) which consisted of female pupils from “Vuk Karadžić” elementary school in Bačka Palanka who were not engaged in any form of organized physical activity except regular 45-min physical education classes, held three times a week (AS = 8.50 ± 1.12 years); (2) Experimental Group 1 (*n* = 51) involving girls that have engaged in a modern dance program for at least 1 year (AS = 8.80 ± 1.22 years); and (3) Experimental Group 2 (*n* = 50) comprising girls that have been involved in a modern dance program for at least 2 years (AS = 8.75 ± 1.05 years). In addition to the dance experience, the participants were also divided by age, with AGE 1 consisting of 103 girls aged 7–9 years (AS = 7.67 ± 0.55) and AGE 2 including the remaining 100 girls aged 9–11 years (AS = 9.62 ± 0.62). The study received ethical approval from the institutional ethics committee at the Faculty of Sport and Physical Education, University of Novi Sad (ethical approval number: 46–06-04/2020-1). All procedures were conducted in accordance with the Declaration of Helsinki and the institution’s ethical guidelines. Written informed consent was obtained from the participants’ legal guardians, and assent was collected from the children before participation. Throughout the study, participants’ safety and wellbeing were prioritized, and appropriate measures were taken to minimize risks during physically demanding activities.

### 2.2 Procedures

The regular training curriculum of the “PLAY DANCE STUDIO” dance club included experimental modern dance programs (1 and 2) focusing on exercises designed to help dancers improve their coordination, lower extremity explosive strength, agility, balance, and flexibility to prepare them for upcoming duties and club goals. The six-month program in focus of this investigation ran from December to May, at which point the last measurements were taken.

#### 2.2.1 Experimental Program 1

Girls who participated in the recreational modern dance program at the “PLAY DANCE STUDIO” dance club formed Experimental Group 1. These girls were at the beginner level, with less than 12 months of dance experience. They were required to attend three weekly training sessions, each lasting 60 min and ranging in intensity from low to moderate. Each class commenced with basic movement patterns and aerobic exercises to appropriate music or games, followed by strength, stretching, and loosening exercises to activate all muscle groups (with particular attention to the major muscle groups that are significant for body posture). The central part of the class was devoted to developing and enhancing motor skills and learning the basics of modern dance, including walking, leaping, jumping, and turning, as well as choreography adapted to the beginner level. The class ended with stretching exercises to loosen the muscles and promote relaxation, as well as to calm the physiological functions.

#### 2.2.2 Experimental Program 2

The girls assigned to Experimental Group 2 were long-term members of the “PLAY DANCE STUDIO” dance club and had been following a competitive modern dance program for at least 2 years. Their training regimen consisted of three 60-min modern dance classes, one 90-min gymnastics class, and one 60-min ballet class. Similar to Experimental Program 1, each dance session commenced with basic movement patterns followed by cardiovascular activities to stimulate all muscle groups through strength, flexibility, and relaxation. The central part of the class was designated for exercises that developed and enhanced motor abilities (interval training, various agility courses), advanced modern dance techniques (walking, leaping, jumping, turns), and choreography adapted to the competitive level of modern dance. As described above, the class ended with stretching activities. The content of gymnastics and classical ballet classes focused on the essential elements of these disciplines according to the dancers’ age and ability level.

For this longitudinal investigation, measurements were performed at baseline as well as at the end of the program, allowing its effects to be evaluated. The pupils in the control group were measured and tested in the school’s physical education hall, while the dance club members were measured and tested in the large hall of the Faculty of Sport and Physical Education in Novi Sad. In each setting, no more than three groups of 20 girls were assessed per day. The assessors were students in their final year of study, as well as graduate-and master-level professors of sports and physical education from the Faculty of Sport and Physical Education in Novi Sad. To ensure their familiarity with the sample and test protocols, all assessors received training prior to their engagement in the study.

### 2.3 Measures

All study participants were subjected to the ALPHA battery of tests, as it is a valid, reliable, feasible, and safe group of health-related fitness tests utilized by the European Union’s public health surveillance system. Depending on the time and resources available for test administration, three slightly different versions of the ALPHA protocol can be adopted, as outlined below (33, 34).

#### 2.3.1 ALPHA evidence-based battery of tests for health-related fitness

The BMI, waist circumference, handgrip strength, standing long jump, skinfold thickness (triceps and subscapular), and the 20-meter shuttle run test are among the evaluations included in this test battery. It has been demonstrated that the chosen tests are related to children’s and adolescents’ present and future health status.

#### 2.3.2 High-priority ALPHA battery of tests for health fitness assessment

A modified version of the above protocol which includes waist circumference and BMI as the sole indicators of body composition.

#### 2.3.3 Extended ALPHA battery of tests for health fitness assessment

This protocol includes the full evidence-based battery of tests as well as 4 × 10 m shuttle run test to evaluate motor abilities.

As the extended ALPHA battery of tests was utilized in this investigation, the measurements included BMI, waist circumference, skinfold thickness (triceps and subscapular skinfold), handgrip strength, standing long jump, 20-m shuttle run test, and 4 × 10 m shuttle run test.

### 2.4 Statistical analysis

All statistical analyses were performed using IBM SPSS commercial software package (SPSS 23.0, IBM Inc., Chicago, IL, United States). Basic descriptive statistical measures were determined for all variables and the Kolmogorov–Smirnov test was conducted to test the data distribution normality. Pre-and post-intervention *t*-tests were performed to assess the difference between the initial and final measurements for each group. To determine the effects of treatment on the transformation of health-related physical fitness in the two experimental groups and facilitate between-group comparisons, multivariate analysis of covariance (MANCOVA) and univariate analysis of covariance (ANCOVA) were applied, considering *p* < 0.05 statistically significant.

## 3 Results

In age group 1 (Table 1), the results of the multivariate analysis indicate statistically significant differences (*p* = 0.00, *F* = 13.14) between the participants of different groups in the physical fitness tests related to health. Further analysis using univariate analysis of covariance revealed that these differences are present in the variables of BMI (*p* = 0.02), Waist circumference (*p* = 0.00), Triceps skinfold (*p* = 0.01), Subscapular skinfold (*p* = 0.00), Standing Long Jump (*p* = 0.00), 4 × 10 m shuttle run (*p* = 0.00), Shuttle run 20 m—number of laps (*p* = 0.00), and Shuttle run 20 m—level (*p* = 0.00).

**Table 1:** Multivariate and univariate analyses of covariance were conducted for the analyzed variables of physical fitness related to health—age group 1.

Variables	Group	AS*	*f*	*p*	η^2^
	C	16.61			
BMI (kg/m^2^)	E1	16.89	4.37	0.02	0.08**
	E2	16.82			
	C	60.64			
Waist circumference (cm)	E1	57.06	22.72	0.00	0.32***
	E2	57.29			
	C	12.30			
Triceps skinfold (mm)	E1	11.10	8.22	0.01	0.14***
	E2	11.03			
	C	8.14			
Subscapular skinfold (mm)	E1	7.09	10.73	0.00	0.18***
	E2	7.28			
	C	123.85			
Standing long jump (cm)	E1	133.27	12.99	0.00	021***
	E2	134.60			
	C	14.96			
4 × 10 m shuttle run test (s)	E1	13.52	50.53	0.00	0.51***
	E2	14.05			
	C	13.13			
Handgrip strength (H/m^2^)	E1	13.49	5.53	0.01	0.10**
	E2	13.62			
	C	17.35			
Shuttle run 20 m—number of laps	E1	21.34	17.82	0.00	0.27***
	E2	19.45			
	C	1.97			
Shuttle Run 20 m—level tests	E1	2.58	18.07	0.00	0.27***
	E2	2.49			
				***F***	***P***
MANCOVA				13.14	0.00

C, control group; E1, experimental group 1; E2, experimental group 2; f, univariate F test; AS*, adjusted mean; p, level of statistical significance of the F test; η^2^, Partial Eta Squared effect size (small*, moderate**, large***); F, multivariate F test; P, statistical significance of the multivariate F test.

After the treatment, we observed an increase in subcutaneous fat in the Abdominal Circumference, Back Skinfold Thickness, and Arm Skinfold Thickness variables in the control group. The experimental group 2 showed the most significant improvement in the Standing Long Jump (AS = 134.60) and Hand Grip Strength (AS = 13.62) tests. Experimental group 1 made significant progress in the Shuttle Run 20 m—number of laps completed (AS = 21.34) and Shuttle Run 20 m—level tests (AS = 2.58). In the 4 × 10 m Shuttle Run test, there was a significant effect after the treatment (*f* = 50.53), with exceptionally high values of Partial Eta Squared indicating the magnitude of the impact (η^2^ = 0.51), and with the best results achieved by experimental group 1 (AS = 13.52).

**Figure 1:**
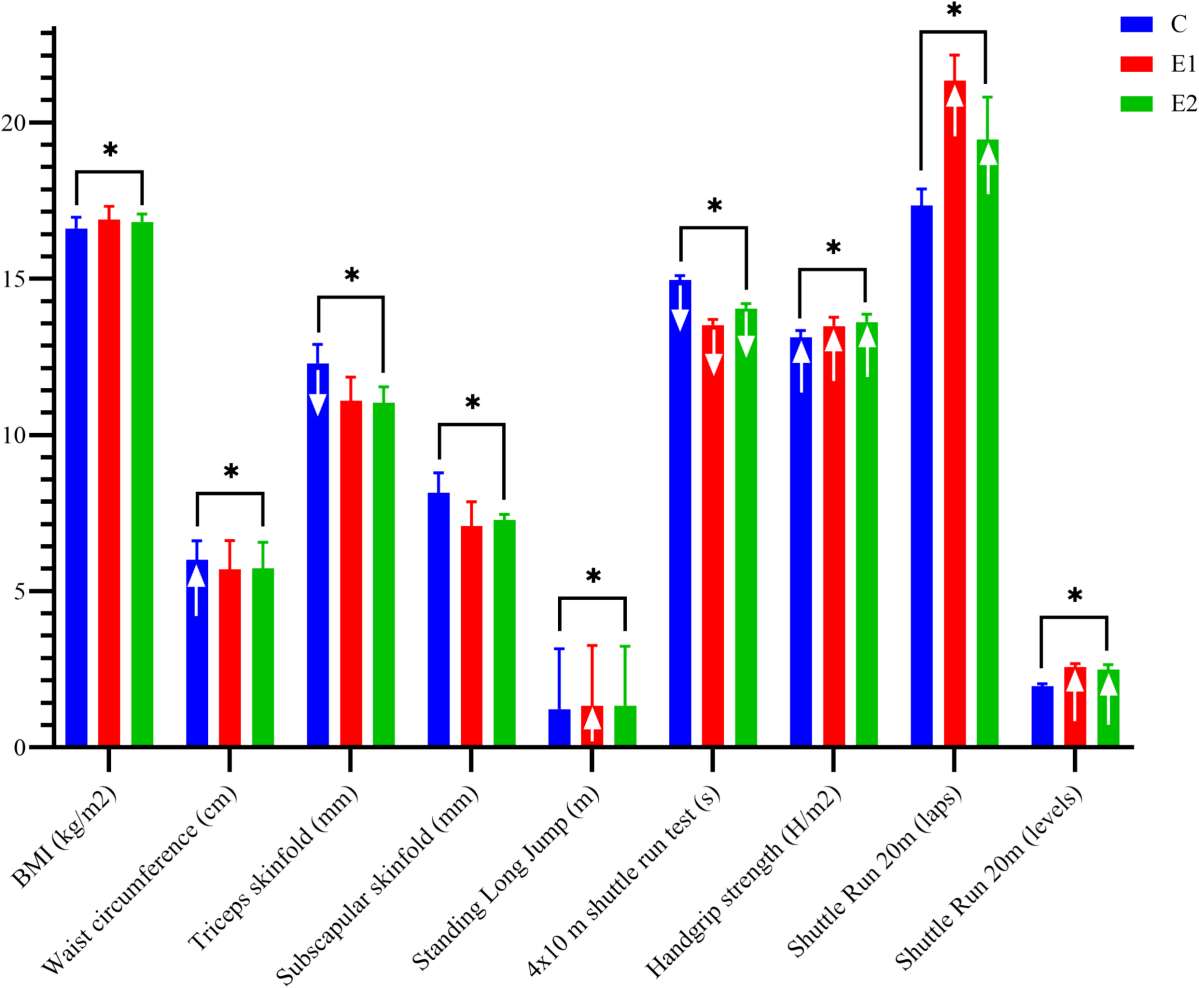
Univariate analyses of covariance and pre to post changes of physical fitness related to health—age group 1. Result are presented in mean and standard error of the mean; ↑ pre to post statistical significant increasement (*p* = 0.05); ↓ pre to post statistical significant decreasement (*p* = 0.05); * level of statistical significance differences of ANCOVA (*p* = 0.05).

Figure 1 presents differences between groups and pre-to post-intervention changes in various measurements. ANCOVA analysis indicates significant differences between groups following the intervention across all variables. Pre-to post-intervention changes were observed in several parameters. The C group showed a significant increase in waist circumference (*p* ≤ 0.05), while triceps skinfold values significantly decreased in the same group (*p* ≤ 0.05). In the Standing Long Jump, E1 demonstrated a significant improvement (*p* ≤ 0.05) after the intervention. All groups experienced significant decreases (*p* ≤ 0.05) in the 4 × 10 m shuttle run test. Furthermore, all groups showed significant improvements in handgrip strength from pre-to post-intervention (*p* ≤ 0.05). In both the Shuttle Run 20 m (number of laps) and Shuttle Run 20 m (levels), E1 and E2 significantly improved their pre-to post-intervention results (*p* ≤ 0.05).

In age group 2 (Table 2), significant effects were observed after the treatment in the following variables: Waist circumference, Triceps skinfold, Subscapular skinfold, Standing Long Jump, 4 × 10 m Shuttle Run, Shuttle Run 20 m—number of laps completed, and Shuttle Run 20 m—level, with a statistical significance of *p* = 0.00. Upon analyzing the results based on adjusted means, it is evident that the control group participants experienced the highest increase in waist circumference and skin folds. Experimental group 1 showed the most improvement in the Standing Long Jump test (AS = 144.81), while experimental group 2 demonstrated the best results in the 4 × 10 m Shuttle Run (AS = 12.79), Shuttle Run 20 m—number of laps completed (AS = 25.92), and Shuttle Run 20 m—level tests (AS = 3.18).

**Table 2:** Multivariate and univariate analyses of covariance were conducted for the analyzed variables of physical fitness related to health—age group 2.

Variables	Group	AS*	*f*	*p*	η^2^
	C	17.44			
BMI (kg/m^2^)	E1	17.73	1.59	0.21	0.03*
	E2	17.84			
	C	64.51			
Waist circumference (cm)	E1	61.89	7.49	0.00	0.14***
	E2	61.81			
	C	13.22			
Triceps skinfold (mm)	E1	11.71	12.78	0.00	0.21***
	E2	11.88			
	C	8.99			
Subscapular skinfold (mm)	E1	7.46	33.70	0.00	0.41***
	E2	7.90			
	C	134.03			
Standing long jump (cm)	E1	144.81	16.87	0.00	0.26***
	E2	141.36			
	C	14.08			
4 × 10 m shuttle run test (s)	E1	13.03	17.83	0.00	0.27***
	E2	12.79			
	C	16.27			
Handgrip strength (H/m^2^)	E1	16.33	0.32	0.72	0.01*
	E2	16.41			
	C	18.76			
Shuttle run 20 m—number of laps	E1	24.29	44.77	0.00	0.49***
	E2	25.92			
	C	2.22			
Shuttle run 20 m—level tests	E1	2.99	39.45	0.00	0.45***
	E2	3.18			
				***F***	***P***
MANCOVA				19.36	0.00

C, control group; E1, experimental group 1; E2, experimental group 2; f, univariate F test; AS*, adjusted mean; p, level of statistical significance of the F test; η^2^, Partial Eta Squared effect size (small*, moderate**, large***); F, multivariate F test; P, statistical significance of the multivariate F test.

**Figure 2:**
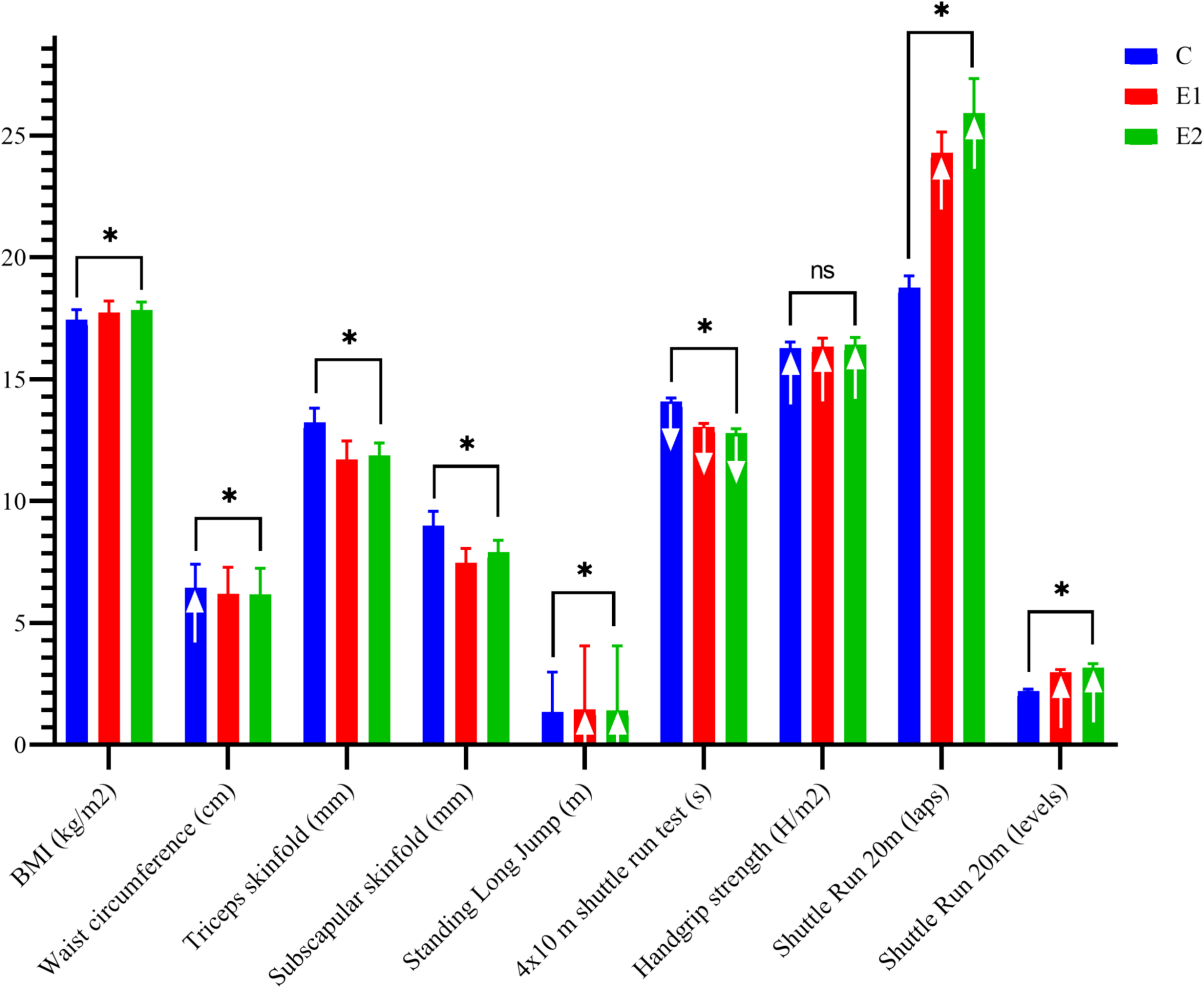
Univariate analyses of covariance and pre to post changes of physical fitness related to health—age group 2. Result are presented in mean and standard error of the mean; ↑ pre to post statistical significant increasement (*p* = 0.05); ↓ pre to post statistical significant decreasement (*p* = 0.05); * level of statistical significance differences of ANCOVA (*p* = 0.05); ns, not significant.

In the age group 2, Figure 2 presents results of ANCOVA and pre-to post-intervention changes in various measurements. Except for handgrip strength, all other measurements showed significant differences between groups according to ANCOVA analysis. The C group showed a significant increase in waist circumference (*p* ≤ 0.05). In the Standing Long Jump, both E1 and E2 significantly improved their performance after the intervention (*p* ≤ 0.05). All groups showed better pre-to post-intervention results in the 4 × 10 m shuttle run test and handgrip strength (*p* ≤ 0.05). However, in the Shuttle Run 20 m (number of laps) and Shuttle Run 20 m (levels), only the experimental groups (E1 and E2) showed significant improvements.

## 4 Discussion

The aim of the present study was to elucidate the effects of modern dance programs in the improvement in health-related physical fitness among younger school-aged children. As this is an insufficiently researched subject, the obtained findings contribute to the existing body of evidence by demonstrating that modern dance programs are an effective means for improving strength, flexibility, and endurance, with the potential to contribute to the promotion of healthier and more active lifestyle.

This topic is of great importance, considering the increasing problem of sedentary lifestyle and the corresponding health issues such as obesity and cardiovascular diseases among children. The results obtained in this study showed that girls who participated in modern dance programs had greater muscle strength, muscle endurance, and aerobic fitness, as well as lower BMI and body fat, after the treatment compared to the baseline as well as relative to the control group. These findings indicate that regular participation in modern dance classes positively affects the physical fitness of younger school-aged children.

The rich and diverse content of the experimental programs in focus of this investigation, along with the proper selection of the means, methods, and appropriate load, initiated a series of anthropological transformations. A large part of the program consisted of intensive exercises to improve coordination, lower extremity explosive strength, flexibility, and balance, which are critical components of dance. Therefore, it is expected that dancers will experience improvements in muscle strength and muscle endurance. However, it was surprising to note a marked increase in the number of laps completed in the 20-meter shuttle run test by Experimental Group 1. Despite the limited space in the dance studios where the program was conducted, owing to a well-planned and carefully implemented modern dance program, a positive effect was achieved in the development of aerobic fitness, which is considered one of the essential components of physical fitness related to health (22) and a direct indicator of children’s health status.

The results of this study indicate significant improvements in several physical fitness components (muscle strength, endurance, and aerobic capacity) among young girls participating in modern dance programs. These findings underscore the value of incorporating dance into school curricula and extracurricular activities as a means of addressing public health issues such as childhood obesity and declining physical activity levels.

Similar findings have been reported by other authors (35–37). For instance, Smith et al. (38) found that children who participated in a dance program showed improvements in cardiovascular fitness and body composition. In an earlier study, Ruiz et al. (22) established that higher levels of cardiorespiratory fitness in childhood and adolescence were associated with a healthier cardiovascular profile in later life. These authors also noted that improvements in muscle strength from childhood to adolescence are inversely related to changes in adipocytes (fat cells). Conversely, based on the assessment of physical fitness in over 1 million Swedish adolescents, Ortega et al. (39) concluded that individuals with lower muscle strength have a higher risk of mortality later in life.

Additionally, as a part of their meta-analysis, Smith et al. (40) investigated the effects of muscle fitness on the health of children and adolescents. The obtained results revealed an inversely proportional relationship between muscle fitness and obesity, cardiovascular diseases, and metabolic risk factors. The authors also found a positive association between muscle fitness and bone health and a moderate to low correlation between muscle fitness and musculoskeletal pain and cognitive abilities. These research findings highlight the importance of developing muscle fitness in children due to numerous health benefits. Specifically, modern dance offers a creative and accessible way to engage children in physical exercise, which could serve as a preventive measure against the onset of cardiovascular and metabolic diseases. By focusing on younger school-aged girls, this research fills an important gap in pertinent literature, given that most prior studies on the effectiveness of physical activity interventions involved other age groups. To address this shortcoming, De Miguel-Etayo et al. (41) proposed a scale for assessing children’s physical fitness, which would allow clubs, schools, and other associations to track participants’ current status or progress more effectively. The scale consists of five levels and children whose physical fitness is low-rated considered an at-risk group, as they are more prone to developing cardiovascular diseases. Therefore, it is of great importance to monitor the physical fitness of children in order to identify potential problems, reduce the risk of various illnesses (42, 43), improve all components of physical fitness, and ultimately improve their health status.

This research therefore adds to the expanding body of evidence supporting the health benefits of creative physical activities, such as modern dance. Unlike conventional sports, dance provides an inclusive form of physical activity that can engage a wider range of children, particularly those who might not be inclined to participate in competitive sports. The obtained findings also underscore the importance of designing programs specifically aimed at younger children, emphasizing that early intervention is key to fostering lifelong habits of physical activity with profound effects on health outcomes.

Available research also indicates that physical fitness testing should be an integral aspect of training programs, as it allows timely evaluation of all human systems’ functional status (9). As the organisztional forms and content of physical education have always depended on the level of development of a particular society and the environment being studied (44), it is not surprising that many countries have recognized the importance of measuring and assessing physical fitness as a part of the formal educational curriculum. Hence, schools can play an essential role in identifying children with lower physical fitness. Regular administration of standardized field tests can also promote physical activity and improve physical fitness (45). Due to its capacity for encouraging good health habits, regular monitoring of physical activity levels and overall physical fitness is a public health priority.

When interpreting the findings reported here, however, it is important to note several limitations to the present study. Specifically, as the control group was recruited from a smaller town and the experimental groups comprised girls from urban environment, their daily habits (including engagement in outdoor play) are likely to differ considerably. As these factors were not taken into account during the analyses, the obtained results might be biased. Another limitation stems from the traditional beliefs and prejudices (46) about dance as a primarily female activity/sport, due to which it was not possible to include a sufficient number of boys in the study. Consequently, the reported results relate to girls only. Finally, the intervention lasted for only 6 months, which may not be sufficient to determine the long-term impact of modern dance on lifestyle and habits.

These and other shortcomings can be addressed through further research. To assess the long-term effects of modern dance programs on children’s physical fitness, health, and overall wellbeing, several longitudinal measures should be considered, encompassing a broad spectrum of physical fitness components, as well as anthropometric, psychosocial wellbeing, and health-related measures.

## 5 Conclusion

Development of motor skills involves more than just physical competence, as it enhances cognitive abilities, including spatial awareness and even academic achievement. Modern dance, in particular, engages several muscle groups and improves fundamental motor skills, including flexibility, balance, and coordination. Young girls can benefit most from this form of exercise because it increases their physical confidence and agility, which promotes greater engagement and better health outcomes. Based on the obtained results, we can conclude that regular participation in modern dance classes, even for recreational purposes only, has the capacity to improve muscle strength, muscle endurance, and aerobic fitness. These findings can be adopted in practice when designing the physical education curricula in schools as well as training programs in dance schools.

## Data Availability

The original contributions presented in the study are included in the article/supplementary material, further inquiries can be directed to the corresponding author.
